# Label‐Free Detection of Virus‐Membrane Interactions Using Surface‐Enhanced Infrared Absorption (SEIRA) Spectroscopy

**DOI:** 10.1002/anie.202502998

**Published:** 2025-06-09

**Authors:** Amelie Teresa Heinen, Saskia Heermant, Daniel Christian Lauster, Stephan Block, Jacek Kozuch

**Affiliations:** ^1^ Physics Department, Experimental Molecular Biophysics Freie Universität Berlin Arnimallee 14 Berlin 14195 Germany; ^2^ Research Building SupraFAB Freie Universität Berlin Altensteinstr. 23a Berlin 14195 Germany; ^3^ Institute for Pharmacy, Biopharmaceuticals Freie Universität Berlin Kelchstr. 31 Berlin 12169 Germany; ^4^ Institute for Chemistry and Biochemistry, Bionanointerfaces Freie Universität Berlin Arnimallee 22 Berlin 14195 Germany

**Keywords:** Influenza a virus, Surface‐enhanced infrared absorption spectroscopy, Tethered bilayer lipid membranes, Viral binding, Viral fusion

## Abstract

Viral binding and membrane fusion are essential steps in viral infection, mediated by viral proteins that bind to host cell receptors and facilitate the fusion between viral and host membranes. Targeting these steps for the development of new antiviral strategies requires methods that enable investigating virus‐membrane interactions under in‐situ conditions, while providing mechanistic insights on a molecular level. Here, we demonstrate the use of surface‐enhanced infrared absorption (SEIRA) spectroscopy combined with tethered bilayer lipid membranes (tBLMs) for the label‐free detection of virus‐membrane interactions, using the Influenza A/X‐31 virus (IAV) as a model. Exploiting the nanometer‐scale surface‐sensitivity of SEIRA, we detect the vibrational fingerprint of IAV's hemagglutinin (HA) glycoprotein, as it specifically binds to sialic acid receptors of the ganglioside GD1a in the tBLM, mimicking the host membrane. Triggering viral fusion via a pH change, we identify structural changes of HA engaging with the host membrane model. Moreover, by constructing the tBLM from deuterated lipids, we utilize the vibrational isotope effect and distinguish between viral and model membrane, providing a basis to track lipid mixing. This approach establishes a powerful tool for spectroscopic studies of the function and inhibition of viral proteins, while still embedded in intact virus particles.

## Graphical abstract

Viral binding and fusion are early steps during viral infection. Surface‐enhanced infrared absorption spectroscopy enables the label‐free tracking of molecular events, as the influenza A virus interacts with a host cell model membrane, providing chemical resolution for viral proteins, viral lipids, and host lipids.

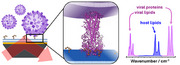

The viral entry process presents multiple early barriers that a virus must overcome before it can infect a cell.^[^ ^1^, ^2^, ^3^, ^4^ ^]^ Initially, the virus binds to the cell surface via interaction with receptors at the host membrane.^[^ ^5^, ^6^ ^]^ Often this involves specific or even multivalent viral protein–receptor interactions which confer cell‐type or even species specificity.^[^ ^7^, ^8^ ^]^ Later, viral uncoating is necessary for the virus to release its genetic information.^[^ ^4^, ^9^ ^]^ In enveloped viruses, which contain a lipid membrane surrounding the viral capsid, this is accomplished by a complex mechanism where viral fusion proteins catalyze the fusion between viral and host membrane.^[^ ^10^, ^11^ ^]^ Even though these steps offer multiple vantage points for antiviral treatment, effective drugs are only available for a limited number of viral diseases.^[^ ^12^, ^13^ ^]^ As such, mechanistic studies of viral binding and fusion carry large potential to uncover new routes to therapeutic strategies.

The advances in fluorescence microscopy‐based single virion binding and fusion assays^[^ ^14^, ^15^ ^]^ have provided important insights into the understanding of binding and fusion of pathogenic viruses, such as influenza, Zika, or SARS‐CoV‐2.^[^ ^16^, ^17^, ^18^, ^19^, ^20^, ^21^, ^22^ ^]^ In these assays, a solid‐supported bilayer membrane or tethered lipid vesicles serve as target membranes mimicking the host cell membrane.^[^ ^15^, ^23^ ^]^ Thanks to the sensitivity of fluorescence microscopy, this enables tracking the dynamic behavior of virus‐membrane interactions, providing information on the fusion efficacy (i.e., the amount of successful fusion events), the extent and kinetics, or the release of content by the virus during the fusion,^[^ ^16^, ^17^, ^18^, ^19^, ^20^, ^21^, ^22^, ^23^, ^24^, ^25^, ^26^ ^]^ importantly, with single virus particle resolution. Despite the relevance of single virion assays in virology, the usage of fluorescence as a readout comes with intrinsic limitations. First, the virus or the target membrane is required to be equipped with exogenous dyes as fluorescent labels, which can impair viral activity or impact physicochemical properties of the membrane, respectively.^[^ ^27^, ^28^ ^]^ Second, despite enabling the detection of single particles, fluorescence does not provide direct molecular information on the underlying biomolecular interactions or structural changes. These drawbacks motivate the development of new methods to complement fluorescence‐based assays.

One approach that can address these drawbacks is surface‐enhanced infrared absorption (SEIRA) spectroscopy^[^ ^29^, ^30^ ^]^ combined with tethered bilayer lipid membranes (tBLMs).^[^ ^31^, ^32^ ^]^ Infrared (IR) spectroscopy is used routinely for mechanistic protein studies^[^ ^33^ ^]^ providing rich information ranging from large secondary structural reorganization^[^ ^34^, ^35^, ^36^, ^37^ ^]^ to subtle changes in electrostatic environments.^[^ ^38^, ^39^, ^40^ ^]^ In SEIRA spectroscopy, a nanostructured Au film further amplifies this information in a surface‐sensitive way.^[^ ^29^ ^]^ Accordingly, based on a plasmonic mechanism, IR signals of molecules within 10 nm to the Au surface are enhanced 10^2^–10^3^ times, lowering the detection limit to the femtomole range.^[^ ^41^ ^]^ The electromagnetic polarization direction further selects molecular vibrations with dipole moment changes perpendicular to the surface (surface‐selection rule).^[^ ^29^, ^30^ ^]^ In addition, the Au film serves as a support to tether the tBLM to the surface‐enhancing 10‐nm region (Figure 1a).^[^ ^29^ ^]^ This provides the unique advantage of selectively monitoring processes at the membrane interface as demonstrated in functional studies of membrane proteins^[^ ^41^, ^42^, ^43^, ^44^, ^45^, ^46^, ^47^ ^]^ or membrane‐active peptides.^[^ ^48^, ^49^, ^50^, ^51^ ^]^ This is in contrast to alternatives like attenuated total reflection IR (ATR‐IR) spectroscopy, which is characterized by a penetration depth of ≥1 µm, therefore detecting interfacial and bulk processes simultaneously.^[^ ^36^, ^37^, ^52^, ^53^ ^]^

**Figure 1:**
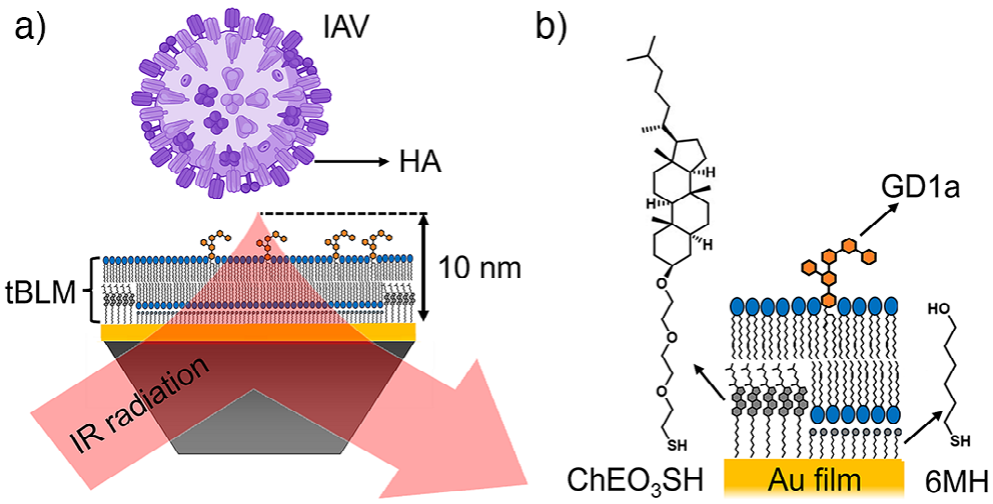
Schematic representation of the combination of SEIRA spectroscopy and tBLMs for the monitoring of IAV‐membrane interactions. a) A tBLM mimicking the host membrane is constructed on an Au film that enables detecting SEIRA spectra with a surface‐sensitivity of 10 nm, for instance, during the binding of IAV via HA glycoproteins. b) The tBLM is based on a phase‐separated self‐assembled monolayer of the cholestanol tether ChEO_3_SH and the spacer 6MH on the Au film, which is covered by a bilayer lipid membrane containing 1 mol% of the sialic acid‐presenting lipid GD1a, serving as attachment factor for IAV.

In this work, we demonstrate the combination of SEIRA and tBLMs as a tool for the label‐free detection of viral binding and fusion events using the Influenza A virus (IAV; X31 strain) as model system. As an enveloped virus, IAV contains an outer membrane, which houses, among others, the hemagglutinin (HA) glycoprotein. This protein binds to sialic acids as specific attachment factors in the host membrane and catalyzes virus‐membrane fusion.^[^ ^10^, ^54^ ^]^ We successfully detect the vibrational fingerprint of the segments of HA as they interact specifically with the tBLM, the latter playing the role of the host membrane. Furthermore, triggering IAV membrane fusion via pH change, we observe relevant viral peptide/membrane interactions and potential lipid exchange events between the virus and the host membrane.

Towards monitoring IAV‐membrane interactions using SEIRA spectroscopy, we constructed a tBLM as a model for the host membrane using POPC with 1 mol% of GD1a as lipids (Figure 1a and b). GD1a is a ganglioside, which presents a sialic acid headgroup as the native IAV attachment factor and is, therefore, often used as specific IAV binding partner in interaction studies.^[^ ^17^, ^55^ ^]^ The tBLM was tethered to the SEIRA Au film via the cholestanol‐headed ChEO_3_SH, which we designed in our previous work (previously referred to as WK3SH) for spectroscopic studies on supported membrane systems.^[^ ^41^, ^48^, ^56^, ^57^ ^]^ Mixing the tether with a spacer molecule, like 6‐mercaptohexanol (6MH), creates a spontaneously phase‐separated self‐assembled monolayer, such that addition of lipid vesicles yields a planar tBLM with free‐floating POPC‐GD1a‐containing lipid bilayer patches of roughly 150 nm diameter (see refs. [41, 58, 59] for characterizations of this type of tBLMs). To ensure that the tBLM has formed as intended, we followed the membrane formation spectroscopically and recorded the membrane capacitance (see Figure S1).

Adding IAV to the supernatant solution (Figure 2a), we monitored SEIRA spectra taking the spectrum of the tBLM as background (Figure 2b; black to purple spectra). We observe the appearance of several spectral features, in particular, the sharp bands at 1659 and 1548 cm^−1^ assigned to protein amide I and II modes, respectively, which indicate a favorable interaction of the IAV particles with the tBLM. Given the ∼10 nm‐surface‐sensitivity and surface‐selection rule of SEIRA, we infer that the detected amide modes reflect a polarized spectrum of portions of the IAV proteins that interact with the GD1a headgroups at the membrane interface, that is, the HA proteins. This is supported by a set of the following observations. Comparing the shape of the amide bands in the SEIRA spectra with a reference spectrum of IAV particles recorded using ATR‐IR spectroscopy (purple spectrum below SEIRA spectra in Figure 2b), we note considerable differences, for instance, in the widths and relative intensities of the amide I and amide II: while in the SEIRA spectra both bands are of similar intensity with an amide I/amide II ratio of 1 (based on peak areas), the ATR‐IR spectrum shows broad features with a considerably larger amide I with a ratio of ∼3.4. This difference can be explained by ATR‐IR detecting a sum of all constituents of the virus (lipids, envelope proteins, capsid, and genetic material as well as enclosed bulk water contribution) in all orientations, while SEIRA shows polarized spectra of the oriented, membrane‐interacting HA proteins (due to surface‐sensitivity and surface‐selection rule). This is supported by SEIRA spectra resembling ATR‐IR spectra when such binding does not occur, which we discuss further below. Moreover, the CH_n_ stretching region (3000 – 2800 cm^−1^) of the ATR‐IR reference spectra of IAV, GD1a, and POPC show dominating peaks at 2924 and 2852 cm^−1^, which are assigned to CH_2_ stretches typical for long alkyl chains of lipids.^[^ ^60^ ^]^ Instead, in the SEIRA spectra, the CH_3_ stretch at 2962 cm^−1^ is strongest as typically observed in protein spectra (grey reference spectrum); the SEIRA spectrum is therefore indicative of the branched alkyl sidechains in HA.^[^ ^60^ ^]^ In addition, we observe changes to the tBLM. In particular in the initial SEIRA spectra, we note a broad band at ∼1600 cm^−1^, which coincides with the GD1a absorption band (orange ATR‐IR spectrum in Figure 2b). Furthermore, in later spectra, a difference peak at 1745(+)/1728(‐) cm^−1^ occurs matching the C═O stretch band of the lipid ester groups (blue ATR‐IR spectrum in Figure 2b). The lipid C═O band itself is composed of two components, that is, non‐H‐bonded and H‐bonded C═O groups at roughly 1740 and 1730 cm^−1^, respectively.^[^ ^61^ ^]^ As such, the SEIRA difference feature indicates a change in the H‐bonding network and/or reduction of lipid hydration upon IAV‐GD1a interaction, as supported by negative water bands (i.e., removal of water from the membrane interface) shown in Figure S2.

**Figure 2:**
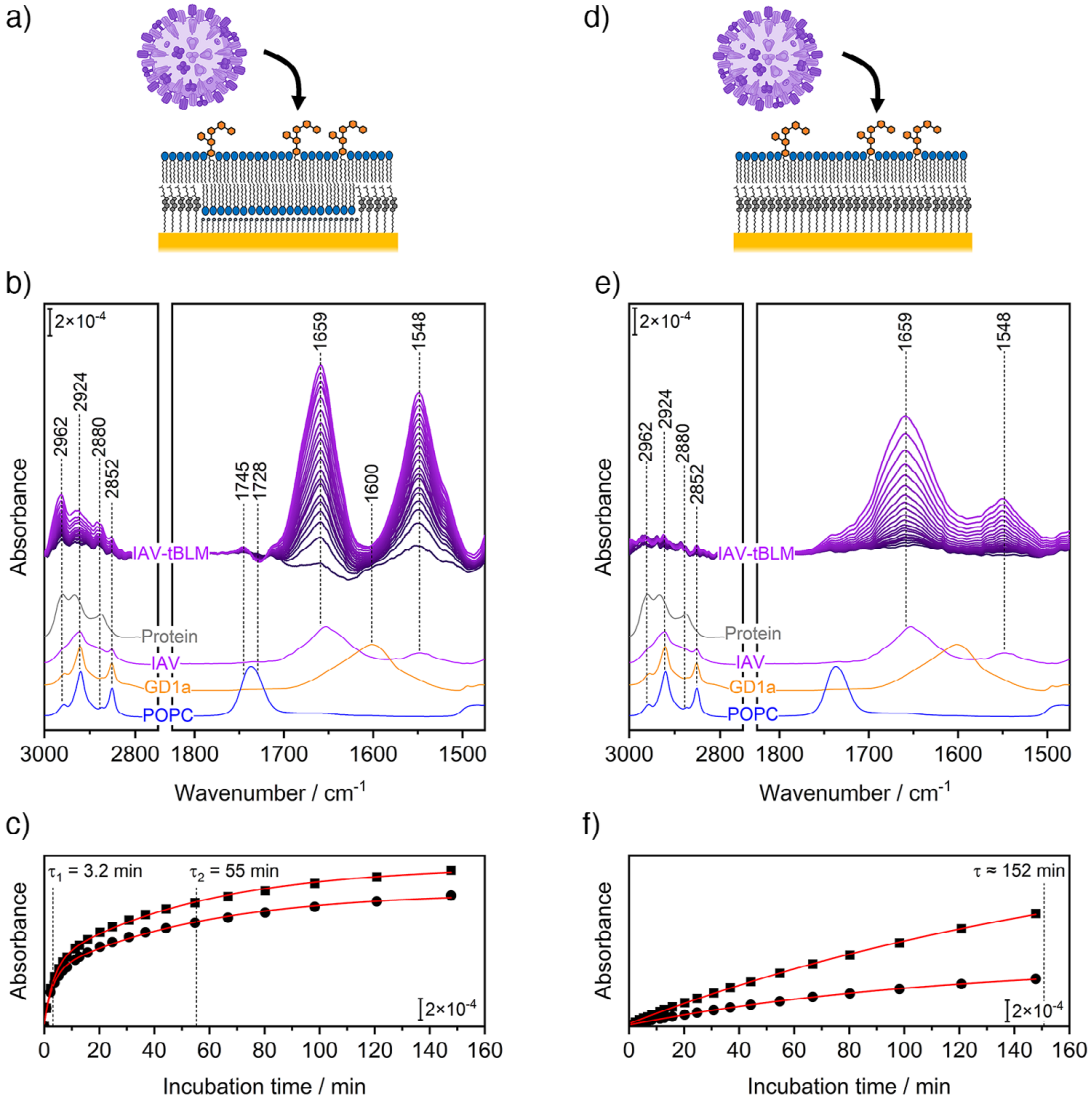
SEIRA spectra of IAV binding to tBLMs with POPC and 1 mol% GD1a as lipids. a, d) IAV binding was monitored on a tBLM containing free‐floating planar bilayer patches on a phase‐separated self‐assembled monolayer of ChEO_3_SH and 6MH (tether and spacer, a) or on a tBLM where a lipid monolayer was supported by a pure ChEO_3_SH monolayer (d). b, e) SEIRA spectra of IAV binding are shown using a color gradient from dark purple to purple for logarithmically averaged time points over a period of 2.5 h (see time points in c and f). ATR‐IR reference spectra for pure components of an arbitrary protein (grey), IAV (purple), GD1a (orange), and POPC (blue) are shown below; the amide region is not shown for the protein due to the high sensitivity of amide bands to secondary structure. c, f) Absorption kinetics of amide I (■) and amide II (●) peak intensities taken from spectra in (b) and (e). Red lines are biexponential (c) or monoexponential (f) fits. Time constants in (c) are similar for amide I and II with 3.2 ± 0.5  and 55 ± 5 min; time constant in (f) is 152 ± 38 min.

Since the tBLM contains patches of free‐floating lipid bilayers and ChEO_3_SH‐supported lipid monolayers (Figures 1 and 2a), we aimed to test to which of these areas virus binding occurred preferentially. Toward this goal, we constructed tBLMs on a pure self‐assembled monolayer of ChEO_3_SH with the same lipid mixture of POPC and 1 mol% GD1a (Figure 2d). Adding IAV, we again observe growing amide bands at 1659 and 1548 cm^−1^, and at an amide I/II ratio of ∼3.1, which now closely resemble the reference ATR‐IR spectrum of IAV. Note that the overlapping amide and water absorption bands contribute to the apparent amide I/II ratios in Figure 2. In Figure S2, we attempt to correct the SEIRA spectra for water contributions which slightly alters the absolute amide I/II ratios, but the difference between the systems in Figure 2a and b remains. Interestingly, very similar SEIRA spectra are detected on tBLMs without GD1a, that is, without IAV's binding receptor (Figure S2). Therefore, the spectra in (Figures 2e and S2) display IAV that *unspecifically* sedimented onto the tBLM, contrasting the markedly different SEIRA spectra in Figure 2b due to *specific* IAV‐GD1a interactions. One can understand the observed difference between SEIRA spectra of *specific* and *unspecific* binding (i.e., Figure 2b vs. e) based on the SEIRA distance dependence. The SEIRA signal is due to an electromagnetic nearfield, which drops with a distance dependence of *d* ^−6^ from the SEIRA Au film (dashed line in Figure 3a).^[^ ^29^ ^]^ As such, most of the SEIRA signal originates from the first 10 nm (red area), where a polarized SEIRA spectrum of oriented HA proteins is detected (square in Figure 3a and b). In fact, analyzing the shape of the amide I band, we obtain accurate relative contributions of secondary structure elements of HA at the membrane interface. The highlighted region in Figure 3b, showing the structure of HA's segments^[^ ^54^ ^]^ interacting with the tBLM, is composed of 46% turn/random, 22% α‐helices and 32% β‐sheets. Fitting the amide I band observed using SEIRA in Figure 2b yields three underlying bands at 1679, 1657, and 1635 cm^−1^ (Figure 3c) that can be assigned to turn/random regions, α‐helices and β‐sheets, respectively, with a very similar composition of roughly 50%, 25%, and 25%.^[^ ^60^ ^]^ It is worth noting that this suggests that the observed spectra in Figure 2b have rather minor contributions due to the neuraminidase protein, a β‐sheet‐rich protein in the IAV envelope, which also binds sialic acids but as part of viral release.^[^ ^62^ ^]^ Contrasting the case of specific binding, at distances >10 nm, the electromagnetic field is considerably weakened but non‐zero. Consequently, the electromagnetic field interacts with all constituents of virus particles at larger distances, which is detected as a spectrum similar to the one obtained from ATR‐IR.

**Figure 3:**
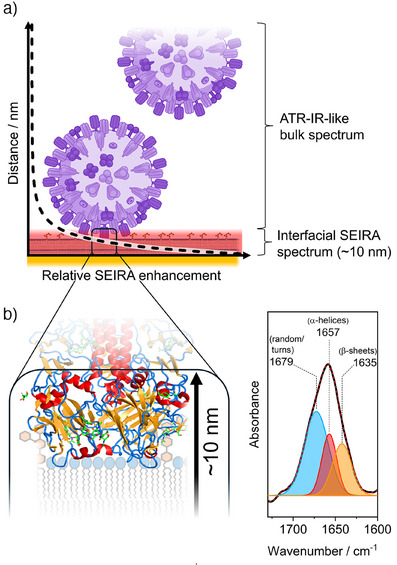
Differences in SEIRA spectra in Figure 2b and d are explained by the SEIRA distance dependence. a) The surface‐enhancing electromagnetic field in SEIRA decays with d^−6^ from the SEIRA Au surface (see dashed line to the vertical distance and horizontal relative SEIRA enhancement axes). A major part of the SEIRA signal is due to the first 10 nm, where a SEIRA spectrum of oriented HA proteins specifically interacting with the tBLM is detected (highlighted region in red and using square box). When IAV is not interacting directly, an isotropic SEIRA spectrum of the entire virus is detected, which resembles a bulk ATR‐IR spectrum. b) Magnification of the membrane‐interacting HA portion (PDB – 6Y5G)^[^ ^54^ ^]^ that is detected using SEIRA; the 10 nm‐region is indicated by the arrow and random/turn regions are in blue, α‐helices in red, and β‐sheets in orange. c) The amide I of specifically interacting IAV (Figure 2b) was modelled using three Gaussians at 1679, 1657, and 1635 cm^−1^, which can be assigned to random/turn regions (blue), α‐helices (red), and β‐sheets (orange), respectively.

The explanation of specific and unspecific binding is also consistent with temporal evolutions of the amide bands. When IAV can bind specifically to GD1a, we observe biexponential kinetics with time constants of 3.2 and 55 min (Figure 2c), which could represent quick initial binding and previously observed mobility of IAV particles, respectively, until efficient multivalent IAV‐GD1a interactions are accomplished.^[^ ^17^ ^]^ In the case when specific binding does not occur, the SEIRA signals increase monotonically with a much slower time constant of approximately 152 min (Figure 2f). Therefore, combining the data from Figure 2, we conclude that SEIRA successfully monitored IAV particles as they bind specifically to GD1a molecules on the free‐floating bilayer lipid patches, but not on the ChEO_3_SH‐supported parts of the tBLM.

An essential step during IAV infection is the pH‐induced fusion of viral and host membrane from the specifically GD1a‐bound state. This process is, at the same time, one of the most complex steps during infection and we sought to examine if the combination of SEIRA and tBLMs can detect the associated structural changes. Mimicking this process, we changed the pH from 7.4 to 5.0 to record the SEIRA difference spectra starting from the final spectra in Figure 2b (see scheme in Figure 4a). We are using difference spectroscopy herein as it is a frequently employed approach to highlight *changes* upon a perturbation.^[^ ^33^ ^]^ These changes are observed as negative or positive signals for species that have disappeared or were created, respectively. In SEIRA they can be further interpreted as molecules that have moved away from or towards the surface‐enhancing region of the first 10 nm to the Au film (negative and positive signals, respectively). After pH change, we observe positive amide I and II bands at 1661 and 1550 cm^−1^ in our SEIRA difference spectra (Figure 4b top), strongly indicating α‐helical protein fragments moving closer to the membrane (note that the presented spectra have been corrected for background pH‐induced changes in absence of the IAV, see Figure S3). At the same time, we observe a negative signal at 1736 cm^−1^ assigned to ester C═O stretches^[^ ^60^ ^]^ of lipid molecules (Figure 4b top). While both viral and tBLM lipids can contribute to this signal, it is reasonable to ascribe this feature to the stronger enhanced tBLM lipids as they move further away from the surface‐enhancing region. This would be in line with the proposed mechanism of pH‐induced HA function. For instance, structural studies have shown that extensive structural changes of HA lead to an anchoring of the α‐helical HA fusion peptide with the host membrane (Figure 4c top → middle).^[^ ^54^, ^63^ ^]^ If the HA fusion peptide engaged successfully, the host membrane is then “pulled” towards the virus. The *positive* HA amide and *negative* tBLM lipid signals are therefore well in line with successful first steps towards virus‐membrane fusion.

**Figure 4:**
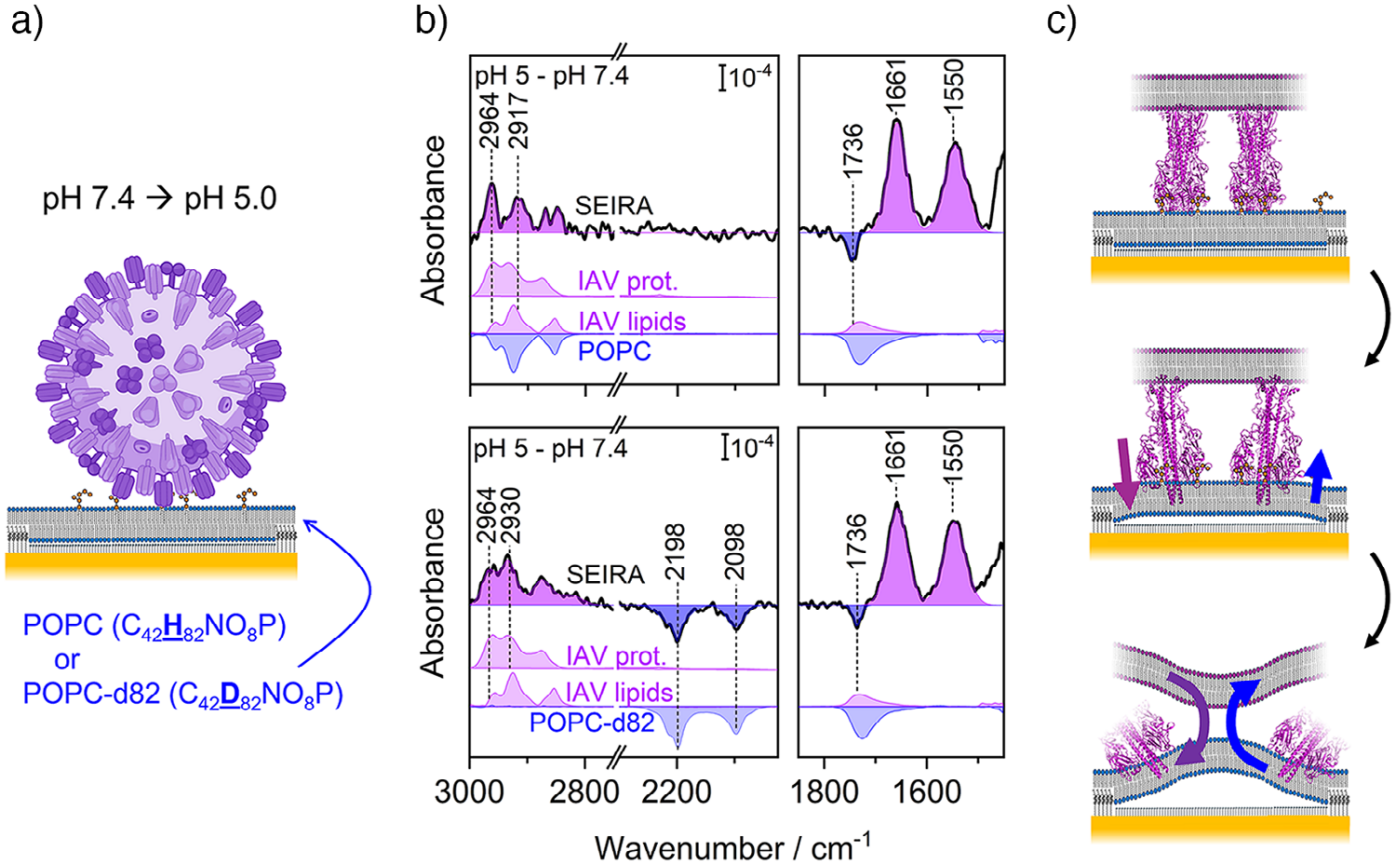
SEIRA difference spectra of pH‐induced structural changes of IAV bound to tBLMs; spectra have been corrected for background pH changes in the absence of IAV (see Figure S3). a) A pH drop from 7.4 to 5.0 was performed with IAV bound via specific interactions to GD1a in tBLM with POPC or deuterated POPC‐d82 as lipids to make use of the vibrational isotope effect. b) “pH 5.0 – pH 7.4″ SEIRA difference spectra (black line) with POPC (top) and POPC‐d82 (bottom) as lipids used in the tBLM; bands highlighted in purple refer to lipids and protein of IAV, whereas bands in blue are assigned to POPC/POPC‐d82 of the tBLM. Light purple or light blue spectra beneath the SEIRA spectra are reference spectra of protein and lipids of IAV or POPC/POPC‐d82 lipids in the tBLM, respectively. Reference spectra are shown with positive or negative intensity according to the interpretation of the SEIRA spectra (i.e., molecules approaching or removed from the SEIRA Au film). See Figure S4 for impedance spectra corresponding to the pH‐induced experiments. c) Schematic representation of structural changes observed in SEIRA difference spectra in (b); protein structures are from PDB ID 6Y5G and 6Y5K.^[^ ^54^ ^]^ (Top → middle) The pH change initiates structural changes of HA, which leads to the anchoring of HA's fusion peptide in the tBLM pulling the bilayer to the virus (purple and blue arrow indicate the motion towards or away from the Au surface observed in b top); this is in line with the positive amide (1661 and 1550 cm^−1^) and negative lipid CO bands (1736 cm^−1^) in (b). (Middle → bottom) When viral and tBLM membranes are pulled together membrane fusion can occur that would lead to mixing of lipids (bend purple and blue arrows indicate the exchange of lipids observed in (b) bottom); such lipid exchange is in line with positive and negative lipid bands (∼2900 and 2100 cm^−1^, respectively) when using POPC‐d82 in (b) (bottom).

For a complete engagement of virus‐membrane fusion process, the viral and target membranes must merge, often observed via lipid mixing or exchange between both membranes.^[^ ^26^ ^]^ In Figure 4b (top), the CH_n_ stretching region shows the strongest peak at 2964 cm^−1^, due to CH_3_ stretches of the HA fusion peptide, which dominate over potential viral or tBLM lipid signals (as indicated below the SEIRA spectra). To separate the spectral contributions due to virus and tBLM, we made use of the vibrational isotope effect and constructed the tBLM using deuterated POPC‐d82. In this way, the tBLM lipids show CD_n_ stretches at 2250 – 2050 cm^−1^, while CH_n_ absorptions of the viral proteins and lipids remain at 3000 – 2800 cm^−1^. Repeating the pH‐induced experiment, we obtain direct indications for lipid exchange. Accordingly, we observe the same positive HA amide and negative lipid C═O stretch signals as the virus engaged in the fusion processes (Figure 4b bottom). In addition, the CH_n_ stretching region (3000 – 2800 cm^−1^) now shows a clear picture of *positive* lipid bands with a dominating CH_2_ peak at 2930 cm^−1^ due to viral lipids; at the same time, we detect a negative CD_n_ stretching region (2250 – 2050 cm^−1^) due to POPC‐d82 lipids of the tBLM. This combination of positive CH_n_ and negative CD_n_ signals directly suggests an exchange of viral and deuterated lipids between both membranes (Figure 4c middle → bottom). We want to note that these spectral markers, however, cannot conclusively confirm a completed fusion process and it is possible that a hemifusion‐like state is adopted.^[^ ^10^, ^11^ ^]^ The opposite intensities also explain why the CH_n_ region in Figure 4b (top) showed a dominating spectrum of HA fusion peptide CH_n_ stretches: in the absence of isotope‐edited lipids, SEIRA signals due to lipids of virus and tBLM have cancelled out.

In summary, the combination of SEIRA spectroscopy and tBLMs provides a new tool to monitor key steps of IAV entry. While it lacks single‐particle (i.e., single virion) resolution offered by fluorescence assays,^[^ ^16^, ^17^, ^18^, ^19^, ^20^, ^21^, ^22^, ^23^, ^24^, ^25^, ^26^ ^]^ SEIRA operates label‐free avoiding the need for exogenous dyes. Furthermore, the approach benefits from the intrinsic molecular structure‐sensitivity of IR spectroscopy and the nanoscale surface‐sensitivity of SEIRA. Notably, SEIRA enables the detection of virus‐membrane interactions while the viral proteins remain embedded in intact virions, eliminating the need for protein isolation as in standard IR spectroscopic studies. As such, we detect specific spectral features of IAV's HA glycoproteins during receptor binding at host membrane model and differentiate between viral and isotope‐edited host lipids during virus‐membrane fusion. In this way, this approach not only complements available viral activity assays, but also our previous SEIRA study of the action and inhibition of the IAV M2 channel within a viral membrane mimic.^[^ ^46^ ^]^ Therefore, in addition to extending this method to studying other viruses and their adaptation^[^ ^64^ ^]^ as well as identifying receptor types and densities,^[^ ^5^ ^]^ the most promising application of SEIRA lies in its potential for testing antiviral compounds that target membrane binding and fusion.^[^ ^12^, ^13^ ^]^ Small molecules or peptides that disrupt hemagglutinin structural rearrangements, such as umifenovir (Arbidol) or rationally designed peptide inhibitors, have been reported to interfere with membrane fusion.^[^ ^65^, ^66^ ^]^ SEIRA spectroscopy can help to provide detailed insights into the structural consequences and further guide the rational design and optimization of fusion inhibitors to enhance their efficacy in antiviral therapy.

## Supporting Information

Additional information is found in the supplementary material: materials and methods; SEIRA and impedance spectra of tBLM construction; SEIRA spectra of IAV incubation; pH‐dependent SEIRA difference and impedance spectra. Additional references are cited in the supporting information.^[^ ^67^, ^68^, ^69^, ^70^ ^]^

## Conflict of Interests

The authors declare no conflict of interest.

## Supporting information



Supporting Information

## Data Availability

The data that support the findings of this study are available from the corresponding author upon reasonable request.
