# Supplementary material for: Label‐Free Detection of Virus‐Membrane Interactions Using Surface‐Enhanced Infrared Absorption (SEIRA) Spectroscopy
Source: Angew Chem Int Ed Engl. 2025 Jun 9;64(31):e202502998. doi: 10.1002/anie.202502998 (PMC12304793; doi:10.1002/anie.202502998)
Supplement: Supplementary file 1 — Supporting Information [file ANIE-64-e202502998-s001.pdf]

# Supporting Information to

# Label-Free Detection of Virus-Membrane Interactions Using Surface-Enhanced Infrared Absorption (SEIRA) Spectroscopy

Amelie Teresa Heinen,[a,b] Saskia Heermant,[a,b] Daniel Christian Lauster,[c] Stephan Block,[b,d] Jacek Kozuch*,[a,b]

a Physics Department, Experimental Molecular Biophysics, Freie Universität Berlin, Arnimallee 14, 14195 Berlin, Germany

b Research Building SupraFAB, Freie Universität Berlin, Altensteinstr. 23a, 14195 Berlin, Germany

c Department for Biology, Chemistry and Pharmacy, Institute for Pharmacy and Biopharmaceuticals, Freie Universität Berlin, Kelchstr. 31, 12169 Berlin, Germany

d Department for Biology, Chemistry and Pharmacy, Institute for Chemistry and Biochemistry, Freie Universität Berlin, Arnimallee 22, 14195 Berlin, Germany

Corresponding author: jacek.kozuch@fu-berlin.de

## Contents

1. Materials and Methods ..... 2
2. SEIRA and Impedance Spectroscopy of tBLMs ..... 3
3. SEIRA Spectroscopy of IAV Incubation on tBLMs ..... 4
4. pH-Dependent SEIRA and Impedance Spectroscopy of IAV on tBLMs ..... 5
5. References ..... 6

# 1. Materials and Methods

*Materials*. 1-Palmitoyl-2-oleyl-sn-glycero-3-phosphocholine (POPC), fully deuterated POPC-d82, ganglioside GD1a (porcaine brain, all Avanti Polar Lipids), 6-mercaptohexanol (6MH), sodium chloride, sodium di-hydrogen phosphate, di-sodium hydrogen phosphate, 2-(N-morpholino)ethanesulfonic acid (MES), and sodium tetrachloroaurate(III) trihydrate (all Sigma-Aldrich) were purchased at highest purity grade available. $ChEO_3SH$ [(β-cholestanoxy)ethoxy)ethoxy)ethanethiol] was synthesized as described previously.[1] The buffer solutions at pH 7.4 (20 mM sodium phosphate and 100 mM NaCl buffer) and pH 5.0 (20 mM MES and 100 mM NaCl buffer) were prepared using Milli-Q-water with a resistance of >18 MΩ cm.

*Surface-enhanced infrared absorption spectroscopy.* SEIRA spectroscopy was conducted using a Bruker Vertex 80v spectrometer using a liquid $N_2$-cooled mercury cadmium telluride detector in a home-built Kretschmann setup at an angle of incidence of 60°. The nanostructured SEIRA Au film was prepared on a trapezoidal Si internal reflection element from a $NaAuCl_4$ solution as described by Miyake et al.[2] All spectra were recorded in a spectral range of 4000 to 1000 $cm^{-1}$ with a resolution of 4 $cm^{-1}$.

*Tethered bilayer lipid membrane construction*. Self-assembled monolayers for the tBLM systems were constructed by adding 200 µL of either (a – for a phase-separated mixed monolayer with ca. 70% $ChEO_3SH$) a 0.5 mM $ChEO_3SH$ and 0.5 mM 6MH solution in 1-propanol or (b – a pure $ChEO_3SH$ monolayer) a 1 mM $ChEO_3SH$ solution in 1-propanol to the SEIRA Au film overnight. After washing with fresh 1-propanol, 1 mL of a 0.5 mg/mL large unilamellar vesicle solution of POPC with 1 mol% GD1a (created via extrusion through 100 nm polycarbonate filters)[3] were added onto the self-assembled monolayers for roughly 3-4 h. Formation of the tBLMs was monitored using SEIRA spectra and the membrane quality was assessed using impedance spectroscopy (0.05 Hz to 100 kHz at a potential of 0 mV vs. Ag/AgCl and an rms-amplitude of 25 mV; Metrohm Autolab PGSTAT204).

*Influenza A virus propagation, characterization and SEIRA experiments*. Influenza A/X-31 (H3N2) virus was prepared as described in Ref. [4]. In brief, the viruses were propagated in embryonated chicken eggs and extracted from the allantoic fluid using several centrifugation and ultracentrifugation steps. Afterwards, the virus was stored in aliquots at -80 °C. The total protein content was determined using the BCA assay (4.9 mg/mL), which corresponds to a number concentration of IAV particles of 71.0 ± 10.8 pM as determined using particle tracking analysis.[4] For the SEIRA experiments, 1.5 µL of the Influenza A/X-31 stock was added into 200 µL of pH 7.4 buffer covering the SEIRA Au film and a series of SEIRA spectra was recorded accumulating 90 s per spectrum (200 scans) as shown in Figure 2 of the main text. After incubation, the system was washed with fresh pH 7.4 buffer. pH-dependent difference spectra were recorded after washing the system three times with pH 5.0 buffer ensuring the pH change; difference spectra of a tBLM without addition of virus were recorded as reference to background changes due to pH. Attenuated total reflection infrared (ATR-IR) reference spectra of pure compounds were recorded in a Resultec ATR setup equipped with a Si internal reflection element. POPC, POPC-d82, GD1a, and amyloid-β (used for protein $CH_n$ region; 1-40, rat, from Echelon Biosciences) where dried onto the ATR-IR Si prism; IAV/X31 was gently dried on the ATR prism from the aqueous solution until no further changes were observed.

# 2. SEIRA and Impedance Spectroscopy of tBLMs

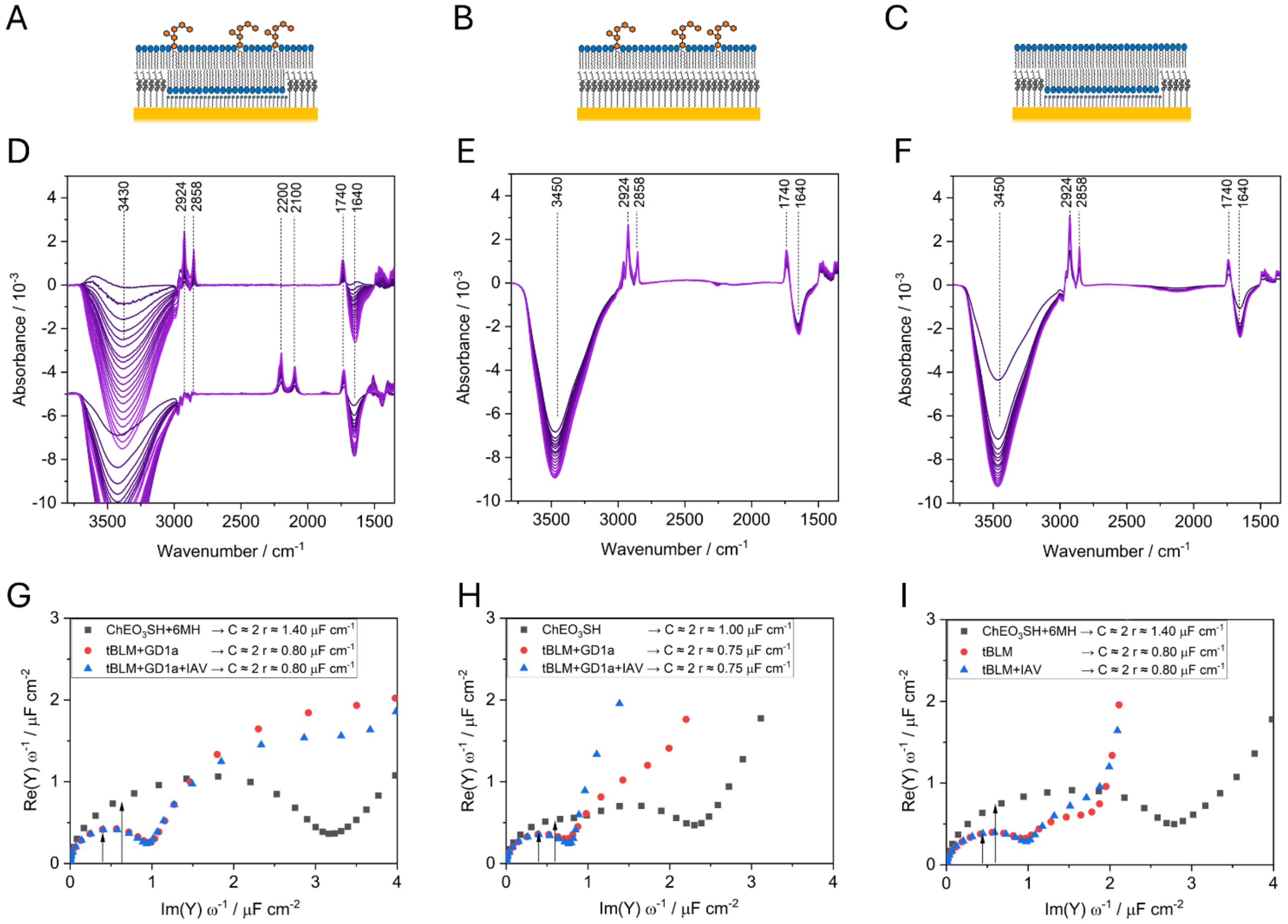


Figure S1. SEIRA and impedance spectra of tBLM systems. A, B, C: tBLMs were constructed (A, D, G) on mixed $ChEO_3SH$/6MH using POPC with 1 mol% GD1a, (B, E, H) on pure $ChEO_3SH$ self-assembled monolayers using POPC with 1 mol% GD1a, and (C, F, I) on mixed $ChEO_3SH$/6MH using POPC only. D, E, F: SEIRA spectra recorded over 3 h of the systems in A, B, and C, respectively. All spectra show characteristic negative bands at ~3400 $cm^{-1}$ and 1650 $cm^{-1}$ due to removal of water from the SEIRA interface. Positive bands at ~2900, 1740 and < 1500 $cm^{-1}$ are due to the adsorption of lipids.[5] In D, spectra are shown when constructing the tBLMs using regular POPC lipids (top) or using deuterated POPC-d82 lipids (bottom) showing the vibrational isotope shift of the $CH_n$ modes at ~2900 $cm^{-1}$ to the $CD_n$ region at ~ 2150 $cm^{-1}$. Residual difference bands at ~2900 $cm^{-1}$ in the bottom spectrum are due to changes in $ChEO_3SH$ during tBLM formation. G, H, I: Impedance spectra of self-assembled monolayers and tBLMs. Capacitances were determined in a model-free fashion from the diameter of the first half-circle representing the capacitance of the entire tBLM (see arrows indicating the radius of the half-circles, which are less resolved for the self-assembled monolayer due to lower resistance than after tBLM formation).[1] The phase-separated monolayers in G and I have a capacitance of ~1.4 µF/$cm^2$ characteristic of a mixed system, whereas the pure monolayer in H has a capacitance of ~1.0 µF/$cm^2$ (see ref. [1]). In each case the tBLM capacitance drops to ~0.8 µF/$cm^2$ as a direct indication of membrane formation with a thicker layer than the monolayers.[1,6–8] See references [2–4] for detailed characterization of this type of tBLM systems.

# 3. SEIRA Spectroscopy of IAV Incubation on tBLMs

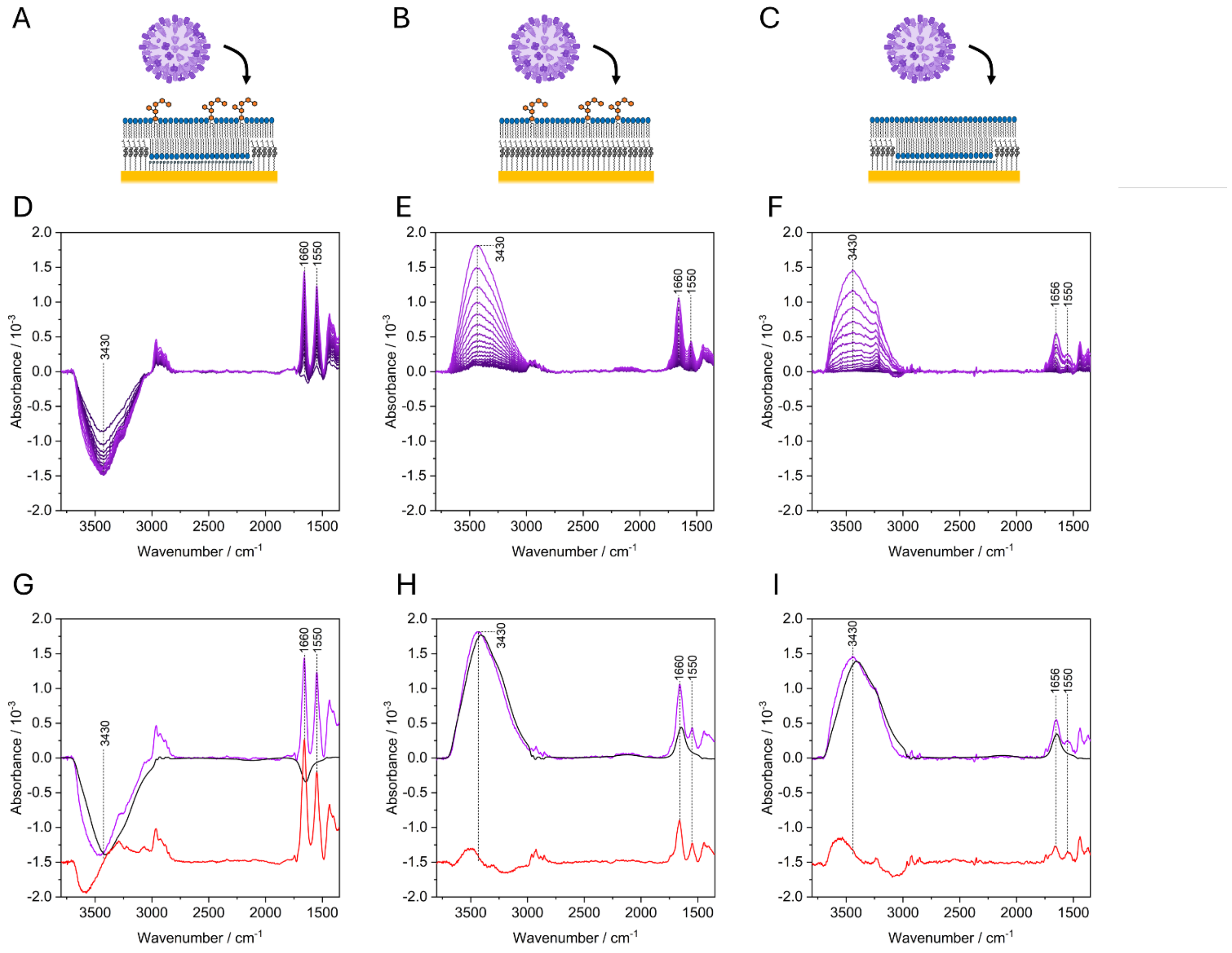


Figure S2. SEIRA spectra of IAV incubation on (A, D) mixed ChEO3SH/6MH-based tBLMs with POPC and 1% GD1a, (B, E) pure ChEO3SH-based tBLMs with POPC and 1% GD1a, and (C, F) mixed ChEO3SH/6MH-based tBLMs with POPC only. D: A negative water band at ~3400 $cm^{-1}$ (and a weaker at ~1650 $cm^{-1}$) coincide with positive protein amide bands at ~1660 and 1550 $cm^{-1}$ (and $CH_n$ stretches at ~2900 $cm^{-1}$), directly indicative of an exchange of water for protein in the surface-enhancing volume within the first 10 nm from the SEIRA interface. E, F: Positive water and protein bands (3400 $cm^{-1}$, and 1660 and 1550 $cm^{-1}$, respectively) excluding specific interaction within the surface-enhancing SEIRA region, instead pointing to unspecific sedimentation (see main text). The slightly weaker bands in F compared to E can be explained by a variation in the surface-enhancing quality of the SEIRA Au film or a slightly smaller viral load. G, H, I: Correction of SEIRA spectra in D, E, and F for contributions due to water absorption in the amide region. Note that the water features at ~3400 and 1640 $cm^{-1}$ can be due to a sum of interfacial and bulk water contributions as the IAV particles are of >100 nm diameter. In the absence of accurate spectra due to these contributions, we use an absolute SEIRA spectrum of water at the Au interface (black), as the best estimate of this sum of interfacial and bulk contributions. The black spectra are scaled to match the feature at ~3400 $cm^{-1}$ and the difference is shown below. The amide I/II ratios change (before and after correction) from 1.0 to 1.3 (G), 3.1 to 2.6 (H), and 3.0 to 2.2 (I); the latter is the most error prone estimate due to the low signal in the original spectra in F.

# 4. pH-Dependent SEIRA and Impedance Spectroscopy of IAV on tBLMs

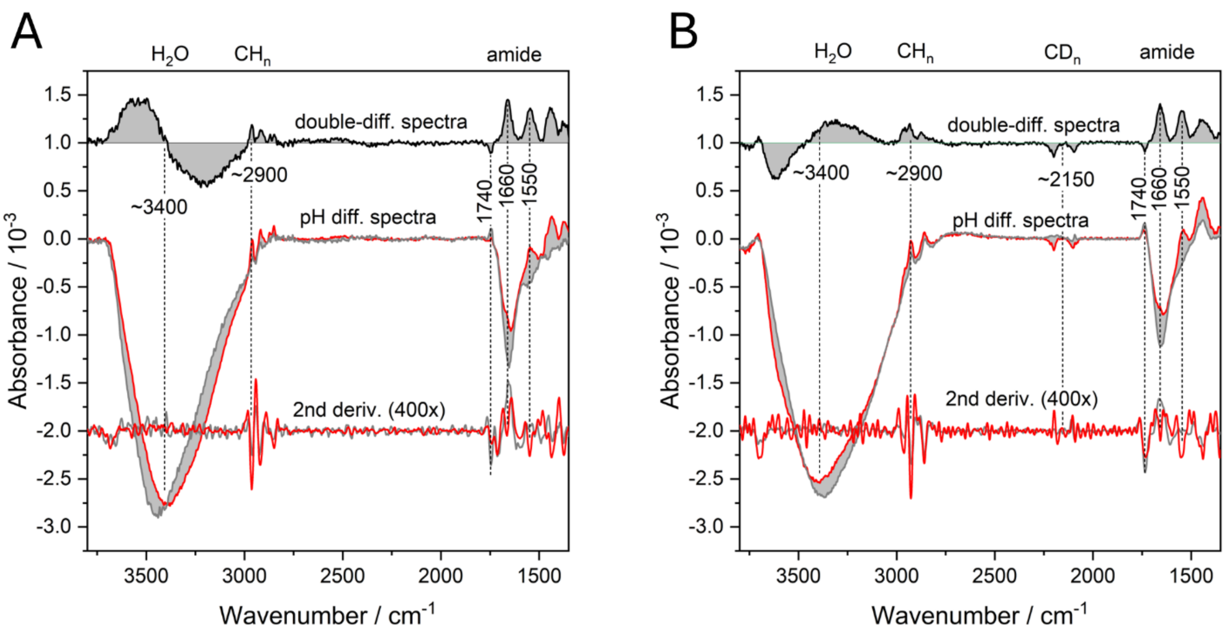


Figure S3. pH-dependent SEIRA (double) difference spectra of IAV specifically bound to tBLMs with POPC (A) or deuterated POPC-d82 (B) upon changing from pH 7.4 to pH 5.0. A, B - middle: Red difference spectra are obtained from IAV bound to tBLMs (similar systems like in Figure S1A,D,G; Figure S2A,D; and Figure 2B in main text). Grey spectra are corresponding pH-dependent difference spectra without bound IAV (tBLM only). In both cases we note negative water changes at ~3400 and 1650 $cm^{-1}$ due to background pH changes. Only in the red spectrum we detect additional changes at ~1660 and 1550 $cm^{-1}$, which can be assigned to protein amide bands.[1,7,9,10] Additional bands are seen in lipid regions, i.e. ~2900, 2200 and 1740 $cm^{-1}$. A, B – bottom: Second derivatives of the middle spectra with similar color coding, also showing the amide bands as negative peaks characteristic for second derivatives. A, B – top: A double-difference spectrum (red-gray) gives the black top spectra (same as used in Figure 4 in main text), highlighting the differences due to the presence of IAV as discussed in the main text. Positive amide bands are in line with the described changes in the single difference spectral; changes in $CH_n$ and $CD_n$ bands are positive and negative, respectively, due to the overlaps of the bands in red and gray spectra. These bands dominate in the red spectra as also visible in the second derivatives (bottom). Note that differences in the shapes of the negative ~3400 $cm^{-1}$ absorption bands of water could originate from slight variations of $ChEO_3SH$:6MH ratio, GD1a content and viral load, as each of the gray and red spectra were recorded in individual experiments. Similar effects are also seen in Figure S1 and S2.

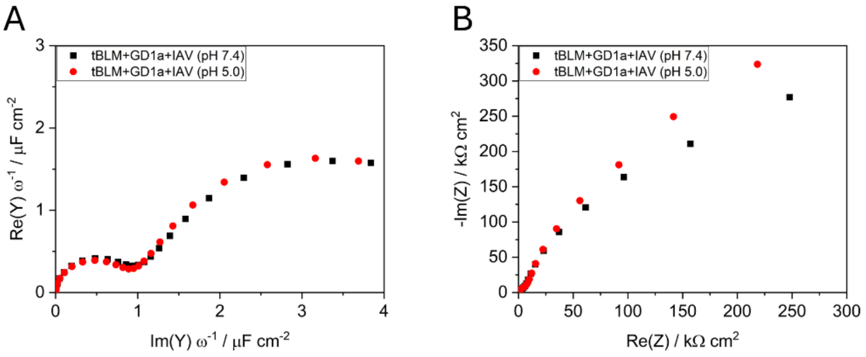


Figure S4. Impedance spectra of pH exchange from 7.4 to 5.0 of tBLMs with 1% GD1a and bound IAV (see scheme in Figure S2A) corresponding to spectra in Figure 4 in the main text and Figure S3. A: Frequency-weighted Cole-Cole plots show that a half-circle with a diameter of < 1 µF $cm^2$ is maintained after pH exchange and the capacitance is slighly lowered at pH 5.0. B: At the same time, the Nyquist plot shows that the resistance of the membrane is overall increased after pH (in line with the lowered connection point between the small and large dispersions in A). A, B: The combination of slightly lowered capacitance and increased resistance can be interpreted as an increased thickness of the tBLM (as observed in multilayered membranes) due to viruses that engaged in the fusion process but remain intact in a hemi-fused form.

## 5. References


[1] S. Wiebalck, J. Kozuch, E. Forbrig, C. C. Tzschucke, L. J. C. Jeuken, P. Hildebrandt, *J. Phys. Chem. B* **2016**, *120*, 2249–2256.

[2] H. Miyake, S. Ye, M. Osawa, *Electrochem. Commun.* **2002**, *4*, 973–977.

[3] E. K. Schmitt, M. Nurnabi, R. J. Bushby, C. Steinem, *Soft Matter* **2008**, *4*, 250–253.

[4] M. Müller, D. Lauster, H. H. K. Wildenauer, A. Herrmann, S. Block, *Nano Lett.* **2019**, *19*, 1875–1882.

[5] L. K. Tamm, S. A. Tatulian, *Q Rev Biophys* **1997**, *30*, 365–429.

[6] J. K. R. Kendall, B. R. G. Johnson, P. H. Symonds, G. Imperato, R. J. Bushby, J. D. Gwyer, C. van Berkel, S. D. Evans, L. J. C. Jeuken, *ChemPhysChem* **2010**, *11*, 2191–2198.

[7] E. Forbrig, J. K. J. K. Staffa, J. Salewski, M. A. Mroginski, P. Hildebrandt, J. Kozuch, *Langmuir* **2018**, *34*, 2373–2385.

[8] A. Erbe, R. J. Bushby, S. D. Evans, L. J. C. Jeuken, *J. Phys. Chem. B* **2007**, *111*, 3515–3524.

[9] J. Kozuch, C. Steinem, P. Hildebrandt, D. Millo, *Angew. Chem. Int. Ed.* **2012**, *51*, 8114–8117.

[10] R. R. Paschke, S. Mohr, S. Lange, A. Lange, J. Kozuch, *Angew. Chem. Int. Ed.* **2023**, *62*, e202309069.
